# Exploring Resilience in UK-Based Domiciliary Care Workers before and during the COVID-19 Pandemic

**DOI:** 10.3390/ijerph192316128

**Published:** 2022-12-02

**Authors:** Warren James Donnellan, Annalise Hirons, Katie Clarke, Christian Muinos, Laura McCabe

**Affiliations:** Department of Psychology, University of Liverpool, Eleanor Rathbone Building, Bedford Street South, Liverpool L69 7ZA, UK

**Keywords:** resilience, domiciliary, carers, COVID-19, qualitative

## Abstract

Domiciliary carers (DCs) provide an invaluable service that enables people living with dementia (PLWD) to remain living in their own homes for as long as possible. We know a lot about the negative impacts of providing domiciliary care and recent evidence suggests that this was exacerbated by the COVID-19 pandemic. However, we know much less about how these DCs manage the stressors associated with their roles. The current study adopts a resilience perspective to identify the resources that DCs caring for PLWD draw on to manage the stress associated with their roles before and during the COVID-19 pandemic. We conducted semi-structured interviews with 19 DCs from across the UK. Data were analysed using a directed qualitative content analysis. Themes included: healthy boundaries; motivation to care; psychological attributes; managing work; and support. The findings have implications for employers and may go some way towards improving DC working conditions, retaining staff, and attracting new DCs in the future.

## 1. Introduction

There are currently at least 850,000 people living with dementia (PLWD) in the United Kingdom (UK), a figure that is projected to reach two million by 2050 [1]. As dementia is a progressive syndrome, its symptoms inevitably worsen over time and PLWD eventually require ‘round the clock’ care and support. Research has indicated that it is the preference of most PLWD to ‘age in place’, that is, to remain living in their own homes for as long as possible. Indeed, the UK’s National Dementia Strategy states that: ‘People with dementia should be facilitated to remain living in their own homes and to maintain existing roles and relationships for as long as possible’ (p. 24) [2].

However, supporting a PLWD to remain at home can be challenging for their primary carers. Dementia care has been associated with increased levels of stress, depression, and anxiety, with 50% of informal carers of PLWD experiencing burnout, burden, and physical health complications, including cardiovascular disease and poor immune responses [3,4,5].

One way of facilitating home care whilst maintaining the health, safety, and independence of PLWD and their carers is through domiciliary care (DC) [6]. In 2014–2015, there were 900,000 people receiving DC in the UK [7]. DCs are a large workforce, with approximately 9400 agencies providing DC and more DCs than residential care workers in England [8]. DCs provide an invaluable service which benefits both PLWD and their informal support networks. In a qualitative interview study of 14 family carers of PLWD, participants praised DCs for their companionship and consistent, personal, reliable, and punctual service which supported the PLWD’s agency and dignity [9].

However, the work of DCs is often physically and emotionally demanding with varied and extensive hours [10]. Client needs are usually complex, and DCs are often inadequately trained in providing dementia-specific care [11]. There is also poor retention and higher staff turnover rates among DCs working in the UK [11]. Research has identified that DCs caring for PLWD experience higher levels of stress and job dissatisfaction and are also more prone to minor injuries and mental health issues such as depression [12,13]. The COVID-19 pandemic brought about a new range of challenges for DCs. The nature of DC meant that social distancing could not always be maintained and shortages of personal protective equipment (PPE) meant that DCs were placing themselves and the people they were caring for at significant risk of contracting the virus [14]. Indeed, the age-adjusted mortality rate of COVID-19 in social care workers (including both DCs and residential care workers) was twice that of healthcare workers employed by the NHS [15].

We know the risks and impacts associated with DC but we know relatively little about any rewards and factors that protect DCs in their important roles. For example, research has shown that adult social care workers report high levels of job satisfaction [16] attributed to the rewarding nature of the role and the perception that they are ‘making a difference’ [17]. Knowing what helps and sustains DCs may go some way towards improving working conditions, retaining the current DC workforce, and potentially attracting new DCs in the future. Employers could also use this knowledge to potentially increase DCs motivations, job satisfaction and resilience, potentially improving outcomes not just for DCs but also for PLWD and their carers [18].

**Figure 1:**
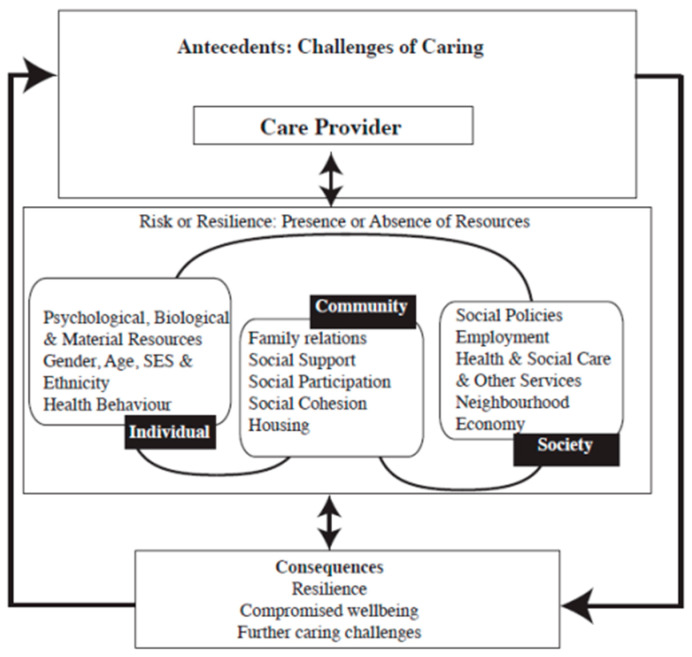
Ecological resilience framework applied to care providers (Windle & Bennett, 2011) [23].

Resilience frameworks are being increasingly used by dementia care researchers to examine the protective factors, assets, and resources carers use to manage the challenges associated with caregiving [19]. Resilience is defined as: “the process of effectively negotiating, adapting to, or managing significant sources of stress or trauma. Assets and resources within the individual, their life and environment facilitate this capacity for adaptation or ‘bouncing back’ in the face of adversity” [20] (p. 163). Resilience is often examined from a trait perspective, identifying various psychological characteristics that are associated with positive outcomes [21]. However, there is growing recognition that broader community and societal contexts also underpin resilience [22] which led to the development of a theoretical ecological resilience framework applied to informal carers [23] (see Figure 1). Unlike other approaches, the framework posits that carers draw on systemic protective factors (community and societal levels) in addition to personal characteristics (individual level) to manage the challenges of caregiving. As such, it is not solely the carer’s responsibility to adapt to stressors but also the community and societal systems in which they live and work. Although this framework has been used to identify factors that support informal carers of PLWD [24,25,26], the extent to which it can be applied to DCs in the context of the COVID-19 pandemic is unclear. Therefore, the current study aims to use the ecological resilience framework to identify the assets and resources that UK-based DCs draw on to protect and sustain them in their roles, both before and during the COVID-19 pandemic.

## 2. Methods

### 2.1. Participants and Recruitment

Recruitment took place between 2018 and 2020. Participants were eligible to take part in the study if they met the following inclusion criteria: at least 18 years old; fluent in English; worked full-time as a DC for at least six months; supported at least one PLWD in their role. We advertised the study via online social media communities for social care workers and through contacting directors of local and national DC agencies who could act as gatekeepers for the research. We asked for expressions of interest to be made either to agency management or to the research team directly. We then shared the participant information sheet with anyone who expressed interest in the study. DCs who met the inclusion criteria and wished to take part in the study were invited to an in-person or remote video interview, where written informed consent would be obtained. We obtained ethical approval from the University of Liverpool prior to the study being conducted (Ref: 1978).

### 2.2. Data Collection

Semi-structured interviews were conducted by A.H., K.C. and C.M. and lasted between 29 and 90 min. Pre-COVID interviews were conducted in person at DC agency offices (*n* = 13) and interviews during COVID were conducted remotely via Zoom or Microsoft Teams (*n* = 6). A.H., K.C. and C.M all had professional experience of being DCs themselves and so this informed the development of the interview schedule and improved rapport with participants. Each interview began with a summary of the research project followed by a series of demographic questions (section A), including: age; gender; place of residence; and experience working as a DC. The interview was then split into three sections. Section B asked participants how they came to work in the social care sector and which aspects of care they found rewarding. Section C covered challenges, asking participants to reflect on which aspects of their role they found most difficult and why, e.g., training, shift patterns, amount of travelling required, and the public perception of social care work. Finally, section D covered resources, asking participants to identify any resources that equipped them to manage their work well, including personal attributes, support networks, and external resources. Each section of the interview encouraged participants to reflect on not just their individual assets but their community and societal resilience resources that they drew on to manage the stress of their roles. Each interview ended by asking participants to provide advice to other DCs and whether there was anything else they would like to share that had not been covered by the interview.

All interviews were audio-recorded using a Dictaphone or the recording function of Zoom or Microsoft Teams. Interviews were then fully anonymised, transcribed, and uploaded to NVivo 12 which was used for analysis.

### 2.3. Data Analysis

We analysed our data using a directed qualitative content analysis [27] whereby a pre-defined coding framework was developed from existing theory, namely the ecological resilience framework [23]. The use of theory in qualitative research provides an analytical lens to interpret the dataset and to enable a comprehensive conceptual understanding [28]. A similar approach has been used previously to apply the same framework to informal carers of PLWD [24,25,26].

The analytical process began by developing a categorisation matrix based on the ecological resilience framework, agreeing on the theoretical definition of the categories, determining coding rules, and coding the transcripts according to the matrix [29]. Three of the authors (A.H., K.C. and C.M.) independently coded all of the transcripts and the remaining authors (W.D. and L.M.) co-coded a subset of the data to ensure rigour. Team meetings were held regularly in which the primary author discussed and compared coding across the research team. A consensus was reached on any discrepancies in coding and all final analytical decisions were revised and agreed upon by all authors.

## 3. Results

A total of 19 DCs took part in the study (13 before COVID-19 and six during COVID-19). Most were female (84%) and aged between 22 and 59 years (mean = 40 years, standard deviation = 11 years). DCs lived and worked across the UK, including the West Midlands (*n* = 7), East Midlands (*n* = 1), Northern England (*n* = 2), North West England (*n* = 6), Southern England (*n* = 1), Mid Wales (*n* = 1), and Central Scotland (*n* = 1). Participants had worked in DC for between six months and 30 years (mean = 8 years, standard deviation = 9 years), worked between 35 and 55 h per week (mean = 42 h, standard deviation = 7 h), and spent between 1.5 to 4 h travelling between calls each day (mean = 2.64 h, standard deviation = 0.80 h). All DCs reported that their agency provided basic induction training, covering areas such as safeguarding, health and safety, safe handling of medication, and manual handling. Some of the DCs had completed additional care qualifications, either in their own time or within their agencies.

### 3.1. Qualitative Findings

The qualitative analysis identified five themes from the data: healthy boundaries; motivation to care; psychological attributes; managing work; and support. Although most of the themes related to the individual level of the resilience framework (e.g., healthy boundaries, motivation to care, psychological attributes, managing work), the community and societal resources (e.g., support) were no less important to DCs. Each theme represents an asset or resource that DCs drew on to manage the challenges associated with their role. Although separate interviews were conducted with DCs before and during COVID-19, the data are synthesised for the purposes of the analysis.

### 3.2. THEME 1: Healthy Boundaries

Most of the DCs spoke about how the line between their work and home lives often became blurred. Key to resilience was the DCs ability to maintain work–life balance. In the quote below, Ann provides a candid account of how she compartmentalises work life and home life:

*There are times, don’t get me wrong, where I do take it home with me. But I have to respect that when I’m at home it’s home life. It does have an impact on it, there’s no two ways about it, but you need to find that line.*

Ann, pre-COVID, DC for 30 years

DCs discussed various ways of maintaining a healthy work–life balance. For some DCs, this meant leaving work at work and switching off completely:

*When you’re at work you switch off everything from home, when you’re at home you switch off everything from work.*

Jasmine, pre-COVID, DC for seven years

Other DCs practiced self-care techniques such as meditation and playing video games to reduce the stress associated with their roles. Self-care was particularly important during the COVID-19 pandemic when workload increased and resources decreased:

*Allowing yourself to have time to do things, to do the things you enjoy the most… Because you need a balance to work in care. You cannot take too much on your shoulders or you’d be making yourself ill.*

Craig, during COVID, DC for six years

With time and experience, the act of maintaining healthy boundaries between work and home life became easier for the DCs. In the quotes below, Anouska and Katie describe the process of leaving work at the door which improved their ability to relax:

*I was going home and thinking ‘ah that person from there…’ and I was saying to my husband, I don’t know whether I can do this job, and it was like that but then now over the years… I go into my house and that’s it.*

Anouska, pre-COVID, DC for 1.5 years

*At first when I started I used to think have I done that right did I sign that… now it’s a lot easier to go home and be like, ‘oh, that’s today done’. But I think once you’ve mastered leaving it all at the door you can go home.*

Vicky, pre-COVID, DC for four years

### 3.3. THEME 2: Motivation to Care

The DCs initial motivations to work in social care varied. For some DCs, the job gave them the flexibility to work around family commitments. Below, Angela describes how her flexible working pattern enables her to look after her children:

*Luckily my hours are flexible and I am really lucky to be able to work around my two boys.*

Angela, pre-COVID, DC for eight years

Interestingly, a number of the DCs had personal experiences of caring for their own family members which inspired them to pursue professional care work:

*I’ve learnt the most about dementia from my own personal experiences.*

Daisy, pre-COVID, DC for ten years

*Well I looked after my Grandad for quite some time, and his care company needed staff so that’s what got me into caring.*

Marie, pre-COVID, DC for 24 years

DCs counterbalanced any negative feelings associated with their work with the enjoyable and rewarding aspects. These aspects were crucial in sustaining the DCs motivation to care:

I did used to think it was wiping a*ses! Like you’ve got no qualifications, nothing… but you do come away smiling 90% of the time.

Eileen, pre-COVID, DC for four years

*Yes, definitely it is rewarding. If you see your clients happy, healthy, that’s the main thing.*

Noreena, pre-COVID, DC for four years

Indeed, many of the DCs felt a sense of honour and pride in their work. This was especially pertinent with the unprecedented challenges posed by COVID-19. It is clear in the following quotes from Lisa and James that a sense of job pride helps them to deal with stressors and gain a sense of job satisfaction:

*Although some days you might be feeling sad, stressed or emotional, you will also feel so good and so rewarded for what you do.*

Lisa, during COVID, DC for 12 years

*It proves how much it means and how much (job pride) can lead your life and job and make it easier dealing with the stress and stuff in this work setting.*

James, during COVID, DC for 1.5 years

*I think working in care and seeing how people react it’s one of the few times in my life when I can actually turn round and say ‘I’m really proud of what I do and how I do it’.*

James, during COVID, DC for 1.5 years

DCs who could see the tangible impact that their care had on their clients and their families were more satisfied and motivated:

*I like helping people… going into somebody’s house and coming out knowing that you have made a difference to their day.*

Daisy, pre-COVID, DC for ten years

*Dementia can be upsetting… so knowing you can calm them down, help them, make them feel more comfortable is brilliant, extremely rewarding.*

James, during COVID, DC for 1.5 years

Over time, DCs developed close professional relationships with their clients. Building these relationships made the job mutually rewarding; the clients were being cared for and the DC felt they were making a difference:

*She began to accept me… I could do a bit more for her, she could… I wouldn’t say remember exactly who I was… but she knew I would be there for her.*

Noreena, pre-COVID, DC for four years

*We’re able to support the families and give them a bit of rest, and they can give us extra insight into who the person was before dementia.*

Angela, pre-COVID, DC for eight years

### 3.4. THEME 3: Psychological Attributes

The DCs possessed a range of psychological qualities that enabled them to manage stress and find positive meaning in their work. Many of the DCs had a stoic mentality; whilst this did not necessarily relieve the pressures of the job it strengthened them to continue caring:

*You have to be strong… when someone is relying on you.*

Louise, pre-COVID, DC for six years

*I think you just get on with it. It’s another day isn’t it so you just do your best!*

Florence, pre-COVID, DC for two years

The DCs demonstrated acceptance which enabled them to remain positive and upbeat, often despite significant challenges. In the quotes below, Louise and Noreena describe how they deal with the hardest part of their work, the death of a client:

*You’ve still got to be that bubbly person even though you’re thinking ‘this person has not got long to live and I will miss them’.*

Louise, pre-COVID, DC for six years

*It’s very hard… but it’s just the circle of life I guess, and you just have to take it like it is, somehow.*

Noreena, pre-COVID, DC for four years

Despite the hardship, some of the DCs were able to bring humour and laughter to their work which was mutually beneficial for themselves but also their clients. In her interview, Louise (pre-COVID) emphasised that ‘good conversation and laughter’ was often just as important as the practical aspects of care. In the quote below, Lisa outlines how her sense of humour helped to reduce the emotional impact of working in care during COVID-19:

*I use humour quite a lot, like, in situations that I find uncomfortable for example. That helps me so much.*

Lisa, during COVID, DC for 12 years

Through working closely with PLWD during the pandemic, Lisa developed a sense of gratitude and appreciation which facilitated personal growth and thankfulness for her own and her family’s health:

*You get to appreciate life more and be more thankful for what you’ve got, and that you and your loved ones are healthy… it’s a very difficult job, but it has helped me grow so much as a person.*

Lisa, during COVID, DC for 12 years

### 3.5. THEME 4: Managing Work

In addition to the above psychological attributes, the DCs described a number of other strategies that helped them to manage the challenges associated with their work. As with the theme of healthy boundaries, time and experience facilitated problem-solving and personal strength:

*Because I’ve done it for so long… I’ve been up against every kind of challenging behaviour or problem you can come across.*

Jasmine, pre-COVID, DC for seven years

*I think the job itself becomes easier with time, because you become stronger and things don’t affect you that much… I think it’s just part of the process really, you just have to adapt and learn how to deal with your own feelings.*

Lisa, during COVID, DC for 12 years

The DCs used every positive or negative experience as an opportunity to learn and gain more knowledge, thus growing as a social care worker. In the following quote, James reflects on his personal and professional growth during the pandemic:

*It (the pandemic) has helped me grow in terms of like… I’ve learnt a lot, I’m more aware. I’m more knowledgeable on not just cornoavirus but things that look like coronavirus but it’s not… like the flu or a cold.*

James, during COVID, DC for 1.5 years

In some cases, it was necessary for the DCs to skillfully adapt their care approach to accommodate the unique needs of their clients. In the quote below, Louise describes how she uses humour to encourage a client who is reluctant to take her medication:

*I have no idea what the tablets do! I just know she needs to take them, so I say ‘well this one could make you beautiful!’*

Louise, pre-COVID, DC for six years

Other DCs take steps to maintain the independence and safety of their clients:

*Leaving everything as their own home, but unplugging the cooker… and putting medicines in a locked box.*

Marie, pre-COVID, DC for 24 years

Finally, the DCs recognised when their clients needed emotional rather than practical support. In the following quotes, Louise highlights the importance of emotional support, maintaining continuity, and a sense of identity for the client:

*It was important that clients felt that DCs cared for them as well as actually provide care.*

Louise, pre-COVID, DC for six years

*If they’ve always worn a suit, let them wear a suit.*

Louise, pre-COVID, DC for six years

### 3.6. THEME 5: Support

DCs drew on a range of supports to help them in their role, both from their own friends and families back home but also colleagues and management within their agencies. The primary source of support for most DDCs was their families. Family support provided physical and emotional strength to the DCs:

*(Family) gives you strength to do what you do.*

Noreena, pre-COVID, DC for four years

*Daughters yeah… well sometimes you’ve got to talk about it, you know, like what’s happened and if you done something nice for someone and the look on their faces, it is good.*

Zinnia, pre-COVID, DC for six months

Family support at home was particularly helpful during the pandemic as DCs were often physically exhausted due to increased job demands and staff shortages:

*I was getting home from my shift and finding out he (husband) had cooked my tea and set up the table with candles… he really wanted me to come from work and have absolutely nothing to do, and I can’t be grateful enough for that.*

Lisa, during COVID, DC for 12 years

DCs also drew on the support of friends outside of work. In the following quotes, Ann and Craig describe the role that their friend networks play in providing emotional support and socialising:

*I’ve got a couple of friends… we’ve been friends a lot of years so we get together and have a little chat, and I know I can pick the phone up to any one of those so it is good. I think it helps having a friend or a number of friends.*

Ann, pre-COVID, DC for 30 years

*I have lots of friends online because I play online games, so I was still able to socialise with them and talk to them, and that felt great, it felt normal… it’s good to just talk.*

Craig, during COVID, DC for six years

Although support from friends and family was highly valued by the DCs, support from co-workers was of special importance. Support from co-workers promoted a special sense of community, camaraderie, and unison. It provided a forum within which the DCs felt more understood and able to share their experiences:

*If you’re friends with them (co-workers) then you’ve got support, because 90% of the time they’re going through the same thing you are.*

Eileen, pre-COVID, DC for four years

*I’ve also got a good set of friends that work with me and we meet outside of work to go for coffee… because if we didn’t meet up for a coffee once a week and get it off your chest, you’ll be banging your head making yourself ill.*

Nastasya, during COVID, DC for eight years

This type of support was particularly important during the pandemic as many DCs spent increased amounts of time at work and may have felt isolated without their colleagues’ support:

*We’ve always been there for each other, not only as friends but as colleagues… and that helps. Having a supportive work environment does help a lot, because you know they’ve got your back.*

Lisa, during COVID, DC for 12 years

Many of the DCs were lone workers, visiting clients without the tangible support of a co-worker. However, DCs made time to socialise online, continuing to build rapport with each other and stay connected when face-to-face contact was not possible:

*We did like game nights and stuff like that through Zoom. So that helped, because you still feel connected to them even though everyone is at home.*

Craig, during COVID, DC for six years

Employer and management support was also critical. DCs who felt that they had the support of their employers felt reassured which made their jobs feel more manageable:

*If there’s any issues with anything (manager) is here you know. She’ll always talk to us about any issues that we have got. But yeah, the support is always there. I’ve never felt like there hasn’t been any support.*

Grace, pre-COVID, DC for 15 years

*I guess I’m just lucky to have them (employers). Because it does make a difference, you know. Knowing they are there if you need them, even like… you might never need them. But if you do, just knowing they are there helps.*

James, during COVID, DC for 1.5 years

Some employers provided the DCs with additional training and support to increase their skillset and coping resources. In the following quotes, Florence and Ann describe how training courses keep them up-to-date with dementia care and what to expect on the job:

*It helps when obviously you’re getting up to date erm like training courses and things like that erm to do like with dementia care or anything really. Just so you know what you’re expecting.*

Florence, pre-COVID, DC for two years

*I think the regular training is excellent, and I do think you need it… I’m just grateful that it’s there to keep you going because otherwise you’d be left behind.*

Ann, pre-COVID, DC for 30 years

Marie was even offered psychotherapy from her employer which she perceived as helpful:

*They’re helpful in every way, they give you counselling or whatever you need… they’re always at the end of the phone.*

Marie, pre-COVID, DC for 24 years

## 4. Discussion

The current study provides some of the first evidence on the resilience of domiciliary care workers both before and during the COVID-19 pandemic. Our qualitative analysis revealed that the line between DC’s professional and personal lives was often blurred, and this was particularly evident during the height of the COVID-19 pandemic. However, the DCs were highly resilient and adaptable. They remained motivated through a sense of acceptance, reward and job pride, and possessed a range of psychological qualities, such as stoicism, positivity, and gratitude, which strengthened them further. DCs who were flexible and adaptable and felt as though they were making a tangible difference to the lives of their clients were better able to manage the challenges associated with their roles. Finally, support from family, friends, and co-workers was important; the latter was particularly important because it created a community of shared experience in which DCs felt well-equipped to support their clients and grow as a care worker.

Most of the evidence to date has focused on the impacts associated with DC work [10,11,12,13,14]. Indeed, our findings highlight some of these challenges, including the work–life balancing act and the emotional impact of providing end-of-life care. However, our findings add to the evidence base by identifying protective resilience resources at the individual level (i.e., healthy boundaries, motivations, psychological attributes, management) and community and societal levels (i.e., support) of the resilience framework [16,17,23]. There were interesting parallels between our findings and those seen in informal carers; both DCs and spousal carers of PLWD demonstrate positivity and gratitude and acquire knowledge and expertise on dementia care over time [24,26]. They both also draw on the support of family and friends [25]. What is different is DC’s motivations to care and the professional supports available to them. This is some of the first evidence to suggest that some resilience resources are universal across informal and formal carers.

Most themes from the current study related to the individual level of the framework, but community and societal levels were also represented and no less important. Indeed, the resilience framework posits that protective resources are non-hierarchical [23]. This supports the ecological approach to carer resilience, whereby interpersonal and environmental resources are highlighted alongside individual demographic or psychological characteristics [19,20,22,23,24]. This has implications for policy and practice; it is not solely the carer’s responsibility to adapt to stressors but also the community and societal systems in which they live and work.

The current study had several limitations. First, although the sample was geographically diverse it may have represented a self-selecting ‘elite’ group of resilient DCs; i.e., those who had time to volunteer in research and willing to share their stories. We therefore may have overestimated the extent of resilience in the DCs and the resources available to them. Second, the sample was unbalanced in terms of pre- (*n* = 13) and post-COVID (*n* = 6) interviews. Although we did not set out to compare pre- and post-COVID experiences, we may have failed to capture the full extent of COVID-19 care experiences. Third, the demographic data could be considered incomplete as we failed to capture details such as ethnicity and socioeconomic status. These data could have been used to purposively sample a more diverse group of DCs and to contextualize the findings more.

Our findings identify potentially modifiable protective factors that could be targeted by policy and practice in the event of future healthcare crises. For example, our findings showed that healthy boundaries were key to resilience. DC agencies should therefore support their employees to take regular annual leave so that they can spend time with their families and friends and prioritise self-care. Furthermore, our findings showed that, with experience, work–life balance became easier and psychological attributes were more developed. Employers could create a ‘buddy system’ whereby new members of staff can learn from more experienced members of staff. Indeed, this peer support was found to be a powerful facilitator of resilience for DCs. Finally, we found that support and training from DC agency management was vital for resilience. Employers should ensure that there is an open channel of communication between DCs and management and that they are responsive in emergency situations when DCs need support. Additionally, by providing regular training courses, employers could ensure that their DCs keep abreast of developments within the social care field and know what to expect on the job which removes ‘fear of the unknown’. These kinds of activities/initiatives could go some way towards improving DC working conditions, retaining staff, and attracting new DCs in the future. Through promoting the resilience of DCs and aiming to increase their job satisfaction and motivations to care, this could also improve outcomes for PLWD and their families [18].

## 5. Conclusions

The current study aimed to use the ecological resilience framework to identify the assets and resources that UK-based DCs draw on to protect and sustain them in their roles, both before and during the COVID-19 pandemic. We found that DCs were highly resilient and adaptable. DCs who maintained healthy boundaries between work and home lives and remained motivated through a sense of acceptance, reward, and job pride were better able to manage the challenges associated with their roles. Additionally, key to resilience was a sense of stoicism, positivity, gratitude, flexibility, and the knowledge that their work was having a tangible impact on the lives of PLWD. Finally, informal support from within and outside the agency meant that DCs felt well-equipped to support their clients and grow as a care worker. The findings support an ecological approach to resilience and may be of interest to DC employers; through knowing some of the resources that DCs draw on to manage job demands, employers can improve job satisfaction, staff retention, and turnover, all whilst indirectly improving the care of PLWD.

## Data Availability

The data presented in this study are available on reasonable request from the corresponding author. The data are not publicly available due to ethical restrictions.
